# Long-Term Biodegradation of Polyacrylamide Gel Residues in Mammary Glands: Physico-Chemical Analysis, Chromatographic Detection, and Implications for Chronic Inflammation

**DOI:** 10.3390/molecules29143247

**Published:** 2024-07-09

**Authors:** Olga A. Legonkova, Naida O. Sultanova, Victoria V. Stafford, Anastasia A. Zavitaeva, Dmitry S. Kopitsyn, Elena R. Tolboeva, Abdul M. Mahmydov, Vladimir A. Vinokurov, Galina A. Davydova, Natalia B. Svishcheva, Katia Barbaro, Julietta V. Rau

**Affiliations:** 1 Vishnevsky National Medical Research Center of Surgery, Ministry of Health of the Russian Federation, Bolshaya Serpukhovskaya Street 27, 117997 Moscow, Russia; oalegonkovapb@yandex.ru (O.A.L.); sultanova@ixv.ru (N.O.S.); v.v.stafford.viev@mail.ru (V.V.S.); anastasia.zavitaeva@mail.ru (A.A.Z.); tolboeva@ixv.ru (E.R.T.); 4abdulmakhmudov@mail.ru (A.M.M.); svishyova.nata@yandex.ru (N.B.S.); 2 Scriabin and Kovalenko Federal Scientific Center, Research Institute of Experimental Veterinary Medicine, Russian Academy of Sciences, Ryazansky Prospekt Street 24. b.1, 109428 Moscow, Russia; 3 Department of Physical and Colloid Chemistry, National University of Oil and Gas “Gubkin University”, Leninsky Prospekt 65, 119991 Moscow, Russia; kopicin.d@inbox.ru (D.S.K.); vinok_ac@mail.ru (V.A.V.); 4 Federal State Institution of Science, Institute of Theoretical and Experimental Biophysics of the Russian Academy of Sciences (ITEB RAS), Institutskaya Street 3, 142290 Pushchino, Moscow Region, Russia; davidova_g@mail.ru; 5 Istituto Zooprofilattico Sperimentale Lazio e Toscana “M. Aleandri”, Via Appia Nuova 1411, 00178 Rome, Italy; katia.barbaro@izslt.it; 6 Istituto di Struttura della Materia, Consiglio Nazionale delle Ricerche, ISM-CNR, Via del Fosso del Cavaliere 100, 00133 Rome, Italy; 7 Department of Analytical, Physical and Colloid Chemistry, Institute of Pharmacy, Sechenov First Moscow State Medical University, Trubetskaya 8, Build. 2, 119048 Moscow, Russia

**Keywords:** polyacrylamide, hydrogel, mammoplasty, breast implants, transformation, degradation products, IR spectrometry, liquid chromatography–mass spectrometry, gas chromatography, differential scanning calorimetry

## Abstract

In the past, polyacrylamide hydrogel was a popular choice for breast augmentation filler, and many women underwent mammoplasty with this gel. However, due to frequent complications, the use of polyacrylamide hydrogel in mammoplasty has been banned. Despite this ban, patients experiencing complications still seek medical treatment. The aim of this study was to investigate the fate of the polymer over a defined implantation period. Biopsies of breast implants were obtained from patients with 23 and 27 years of post-mammoplasty. These biopsies were meticulously purified from biological impurities and subjected to analysis using IR spectrometry, liquid chromatography—mass spectrometry, gas chromatography, and differential scanning calorimetry. The findings revealed the presence of polyacrylamide hydrogel residues, along with degradation products, within the infected material. Notably, the low-molecular-weight degradation products revealed via gas chromatography are aggressive and toxic substances capable of inducing chronic inflammation. This study sheds light on the long-term consequences of polyacrylamide hydrogel implantation, highlighting the persistence of harmful degradation products and their role in exacerbating patient complications.

## 1. Introduction

**Figure 1:**
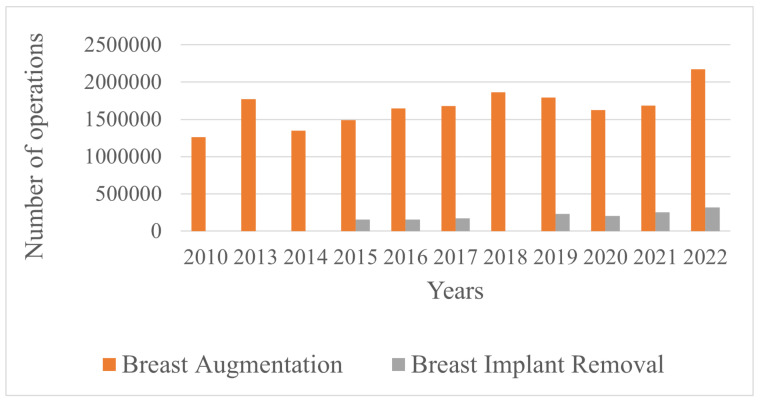
The number of breast augmentation and removal operations according to the data from the International Survey on Aesthetic/Cosmetic Procedures (ISAPS) [1].

One of the most popular plastic surgeries in the world is breast augmentation [1]. It is performed both for breast reconstruction after removal of cancerous tumors or transgender operations [2] and for esthetic purposes. According to estimates from the International Society of Aesthetic Plastic Surgery for the period of 2010–2022, an increase in the number of breast augmentation surgeries is visible (Figure 1). At the same time, more than half of the patients are aware of possible infectious complications after surgery and the likelihood of leakage/damage to the implant [3]. The popularity of breast augmentation surgery is explained by psychological reasons [4]. The procedure has a positive effect on human self-perception and social interactions. However, after surgery, patients often return to the hospital complaining about the problems with their implants. Some of these complaints are related to psychological reasons [5], but many have legitimate physical causes. Most patients complain of fatigue, brain fog, joint pain, anxiety, hair loss, depression, rash, autoimmune diseases, inflammation, and weight problems [6].

Polyacrylamide gel (PAAG) injections into the breast began in the 1980s and ended with a ban on the use of PAAG in many countries in 2006. Polyacrylamide gel contains 2.5–5% polyacrylamide in water [7]. PAAG-based implants caused complications such as pain, breast solidification, breast deformity, lump formation, migration, and gel leakage [8]. Since the 2000s, methods for diagnosing problems with breast implants have been developed [9,10], and complex treatment methods have emerged [11,12,13]. However, despite the ban on PAAG, people who had undergone breast augmentation surgery with PAAG did not remove the toxic implants. Moreover, PAAGs have reappeared on the market despite their bad reputation [14].

Operations to remove breast implants are carried out at our medical center. In this article, we will consider the consequences of breast contouring using polyacrylamide gel, focusing on the degradation of the polymer in the human body. The negative consequences known as polyacrylamide mammary syndrome (PAMS), or “gel” disease, continue to pose a significant problem [15]. A considerable number of individuals who underwent injection contour correction of mammary glands urgently seek hospital care due to the development of late purulent-inflammatory complications, including gel fistulas, infiltrates, and recurrent purulent mastitis. There are publications on the destruction of PAAG under ultrasound and chemical agents [16], as well as its decomposition to acrylamide in vitro in the presence of a hydroxyl radical [17]. When studying tissue reactions in an in vivo rat model (*n* = 80) to a polyacrylamide gel filler, it was found [18] that the polyacrylamide gel filler exhibits high biological activity, undergoing cellular infiltration and integration into tissues. Some publications claim that polyacrylamide hydrogel is well tolerated by the breast and does not cause severe fibrosis, pain, or shrinkage of the gel’s capsule [19]. There are numerous reports in the literature about complications following the implantation of polyacrylamide gel [20,21]. However, there is no information about what happens to the polymer over the long term following implantation. The literature that we reviewed contains information about PAAG implants being inserted into breasts for up to 20 years, but we found no information about their prolonged presence beyond this period.

The purpose of the work stems from the relevance of this study: to investigate surgically removed tissues and to determine the contribution of polyacrylamide gel degradation to inflammatory processes. This will be achieved by examining biopsies of breast explants using histochemical analysis, as well as analyzing samples purified from biological impurities through IR spectrometry, high-performance liquid chromatography, chromatographic-mass spectrometric analysis, and differential scanning calorimetry.

## 2. Results and Discussion

**Figure 2:**
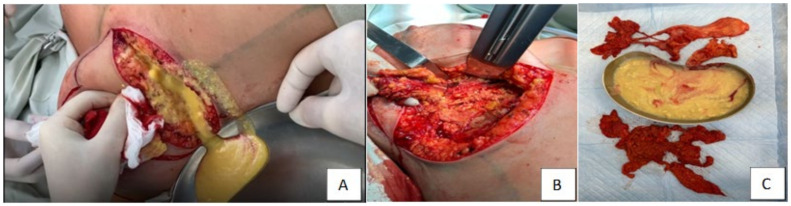
The surgical picture for mammary syndrome: (**A**) removal of gel masses; (**B**) wound surface; (**C**) gel masses with admixture of blood and fragments of removed tissue of mammary glands and large pectoral muscles.

The surgical procedure for addressing mammary syndrome is shown in Figure 2. When dissecting soft tissues, abundant yellow discharge with small inclusions was observed. After the rehabilitation of the wound cavity, it was found that the pathological discharge was contained within a connective tissue capsule with a tendency to migrate. Rounded inclusions, the so-called “geleoma”, were found in the adipose tissue and breast tissue. Similar changes were observed in the connective tissue capsule surrounding the pathological process. The study focused on the gel masses removed after breast surgical interventions. The following samples were investigated:

- MG-23: Gel implanted in the mammary glands for 23 years.
- LB-27: Gel from the left breast implanted for 27 years (contains dense infiltrate inclusions).
- RB-27: Gel from the right breast implanted for 27 years.

**Figure 3:**
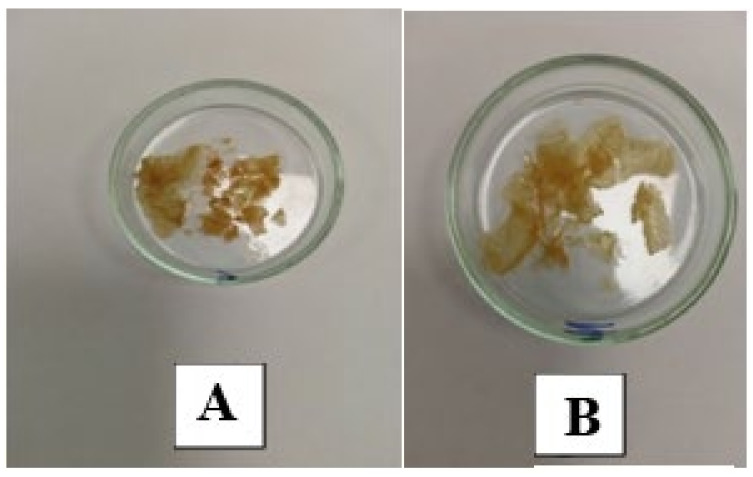
Examples of washed and dried samples: (**A**)—LB-27; (**B**)—RB-27.

The target products (PAA residues) had a light yellow color, as shown in Figure 3. The dry residue content of the removed gel samples LB-27 and RB-27 was 2 ± 0.2 wt%.

**Figure 4:**
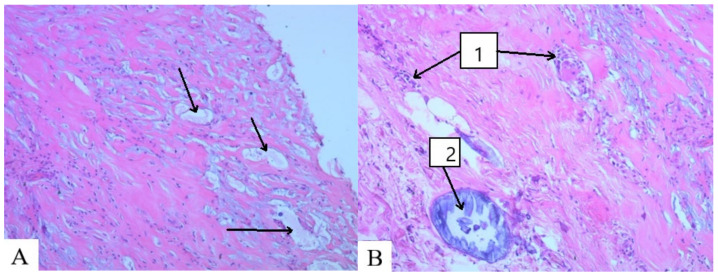
Capsule of the pathological focus, MG-23: (**A**) fragments of a foreign body, formation of voids (arrows); (**B**) 1. lymphoid cell infiltration; 2. petrification site. Hematoxylin and eosin ×100.

**Figure 5:**
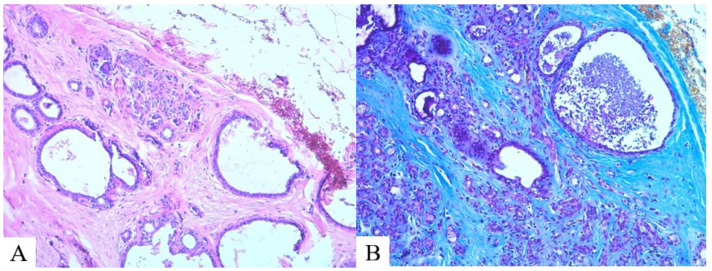
Breast parenchyma, MG-23: (**A**) cystic cavities (hematoxylin and eosin); (**B**) massive bundles of dense connective tissue (Mallory staining ×100).

Histological examination of tissues surrounding the polymer implant is considered the gold standard among modern methods for investigating the degree and nature of inflammation following the extraction of polymer surgical implants. We observed that the capsule of MG-23 is composed of dense connective tissue fibers. Foreign body veins are visible within the fibers, exhibiting a slightly basophilic color. The formation of voids is noticeable, occasionally containing an amorphous substance, with areas of petrification and clusters of lymphoid cells being prominent (Figure 4). There are many cystic, expanded cavities located in the parenchyma of the breast (Figure 5A). When stained according to Mallory (Figure 5B), extensive bundles of dense connective tissue and deformed glands are clearly visible.

The identified process is characterized as mastopathy due to aseptic necrosis. The foreign substance migrates within the tissues, causing active growth of connective tissue, leading to the presence of an aseptic inflammatory reaction and deformation of breast structures. The connective tissue of MG-23, in turn, tightens the surrounding tissues, which leads to a disruption of lymph and blood flow. In addition, it promotes the expansion of glands. All of this contributes to tissue stagnation, leading to the development of edema, inflammatory reactions, and necrosis of the surrounding tissues.

**Figure 6:**
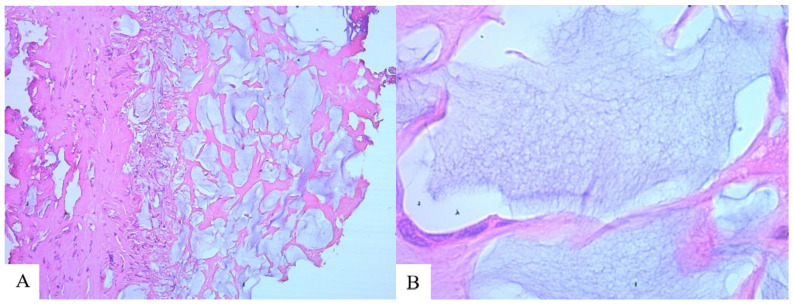
Capsule of the pathological focus, LB-27: (**A**) Fragments of a foreign body, between the fibers of connective tissue (hematoxylin and eosin ×100); (**B**) Structure of a foreign body (hematoxylin and eosin ×630).

**Figure 7:**
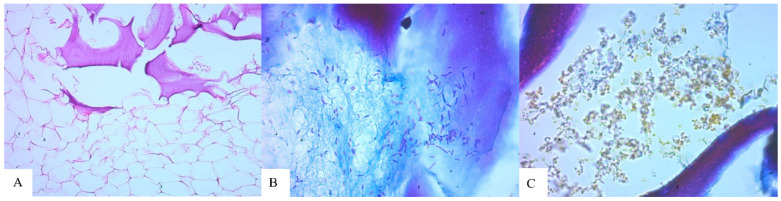
LB-27: (**A**) Next to adipose tissue; (**B**) Large rod-shaped bacteria in/on a foreign body; (**C**) Fine particles and coccoid and small rod-shaped bacteria between parts of a foreign body. Hematoxylin and eosin, (**A**) ×100; Mallory staining, (**B**,**C**) ×630.

The LB-27 sample (Figure 6) is represented by a dense connective tissue capsule with no clear boundaries. However, the interface between the tissue and the foreign body is identifiable, with voids containing a foreign, “foamy” substance (presumably gel) clearly visible. At a higher magnification, the porous structure of the foreign body within the newly formed cavity is visible. Fibroblasts are located along the edge of the cavity. Figure 7 depicts an amorphous foreign body adjacent to intact adipose tissue. Within the structure of the foreign body, numerous rod-shaped bacteria and fine particles consisting of both rod-shaped and coccoid bacteria were detected between the strands of the substance.

**Figure 8:**
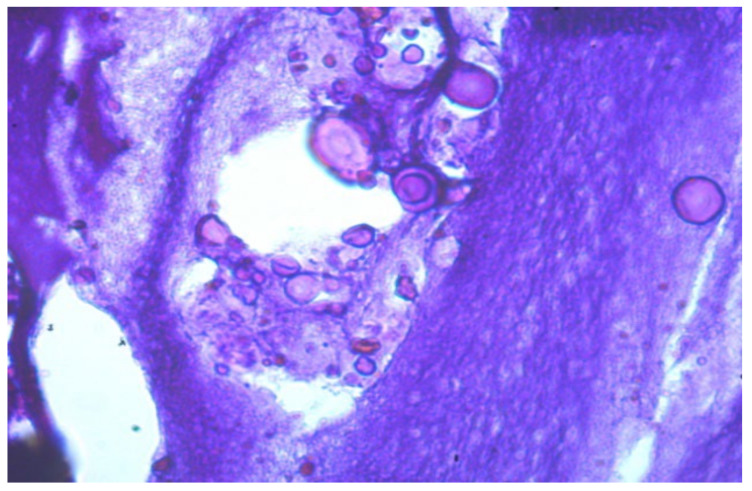
RB-27: The foreign body is presented in an amorphous state, in the form of spheres and small particles. Mallory staining, ×630.

The histoarchitectonics of the pathological process in RB-27 is similar to that observed in LB-27. It is worth noting that the gel (foreign body) in the sections appears to have aspherical shapes (Figure 8). Furthermore, histological examination of pathological breast samples revealed areas of petrification with necrosis of adjacent cells of adipose and muscle tissue. These observations suggest the migration of the foreign body into the surrounding tissues.

Thus, the histological appearance of all examined samples, regardless of the implantation period [22], is similar and is characterized by basic features such as the presence of:

- Foreign substance (gel) migrated in the tissues, provoking the active growth of connective tissue;
- Aseptic and septic inflammatory reactions;
- Sections of round-shaped petrifications with clear contours;
- Lymphoid cell reaction, the presence of voids and necrotic masses, and the formation of cystic cavities;
- Pronounced deformation of adipocytes;
- Muscle tissue with extensive necrosis and effusion of the protein component;
- Numerous mast cells;
- Isolated, giant Pirogov–Langhans cells surrounded by lymphocytes and macrophages.

PAA gel (or its residues) is found in two forms: dense structures inside the “heleomas”, or a less dense form, like liquid, diffusely located in tissues. PAA gel contains both cross-linked chains and free chains. Since patients are unaware of the brand of their implants, the cross-linking agent in PAA remains unknown. For example, the cross-linking agent may be N,N′-Methylene bis(acrylamide) (BIS). During the use of the implant, the cross-linked chains may degrade. Linear chains of macromolecules also undergo various transformations, over time. PAA is a well-known polymer capable of undergoing a number of chemical transformations forming ionic derivatives, branched and cross-linked polymers, as well as conjugates with various macro- and low-molecular components of biological media [23,24,25]. In general, chemical transformations of PAA may include the following reactions:

1. Rupture of cross-linked chains. Nucleophilic effect of protonated solvent on cross-linked sections of macromolecules:

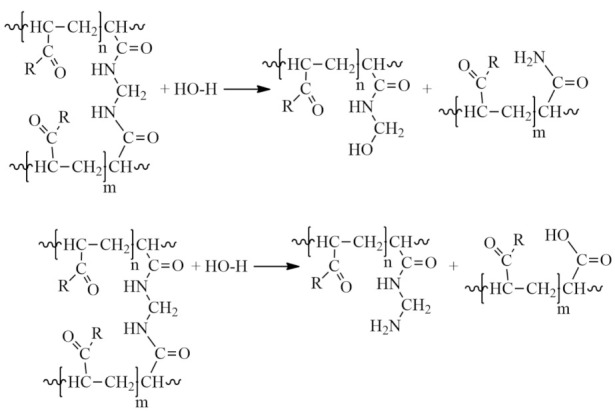

2 Partial hydrolysis under slightly acidic conditions:

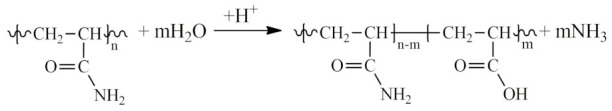

The degree of partial hydrolysis under acidic conditions can reach a significant level. Moreover, when the degree of conversion reaches 3–5%, hydrolysis in an acidic medium is auto-catalytically accelerated.

3 The formation of partially or completely insoluble products due to the imidization reaction in acidic and slightly acidic media, leading to the formation of cyclic and three-dimensional spatial structures:

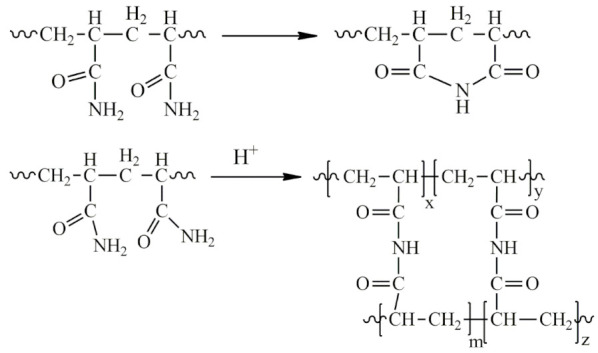

4 Hydrolysis of the amide group in a slightly alkaline medium under the action of hydroxides and carbonates:

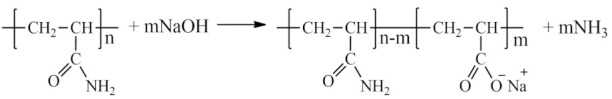

Alkaline hydrolysis of PAA results in the formation of macromolecules of acrylamide copolymers with acrylic acid salts, exhibiting a statistical distribution along the polymer chain without block structures. The maximum degree of hydrolysis of PAA in alkaline conditions does not exceed 70% due to the reduced reactivity of amide groups blocked by neighboring carboxylate groups, as shown in the scheme:

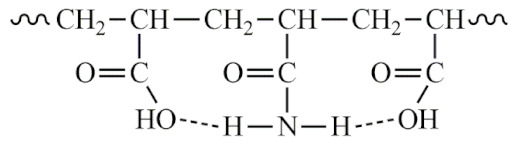

5 Aminomethylation of PAA with aldehydes (formaldehyde, acetaldehyde, 5-hydroxymethylfurfural, malondialdehyde, fatty aldehydes, etc.) and primary and secondary amines in a slightly alkaline medium to form an aminomethylated polymer according to the scheme:

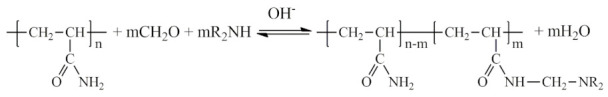

6 The interaction of PAA with endogenous and exogenous aldehydes in acidic and slightly acidic media results in the formation of intramolecular cross-linking:

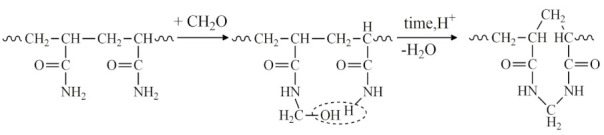

In this case, intermolecular cross-linking can also occur, resulting in the formation of three-dimensional structures (-CONH-CH_2_-NHCO-):

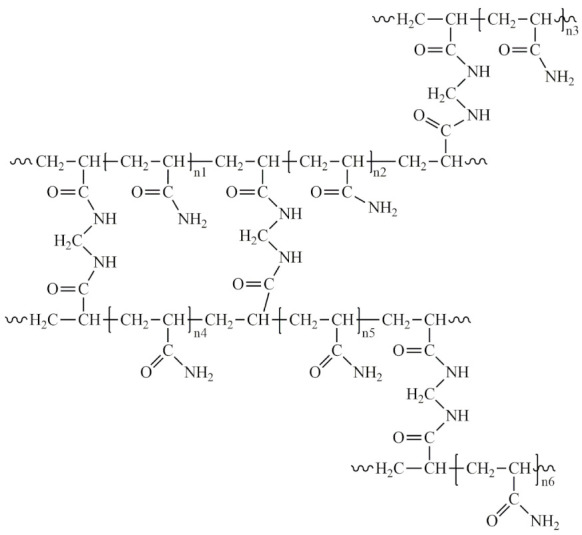


**Figure 9:**
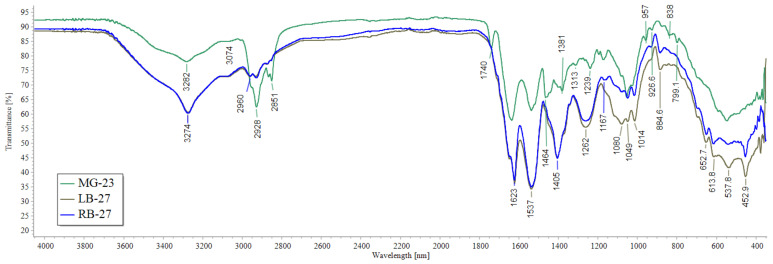
IR spectra of MG-23, LB-27, and RB-27 samples.

The structure of the samples was analyzed using IR spectroscopy. The spectra were interpreted based on characteristic frequencies using the IR spectra library in the LabSolutions IR software (version 2.2). Table 1 lists the types of vibrations used to identify the spectra [26]. The IR spectra of the studied samples are similar to each other (see Figure 9): there are characteristic lines of primary, secondary, and tertiary amines and amides; the spectra mainly contain characteristic vibrations of the -O-NHR and -C(O)- ether groups. Thus, the IR spectroscopy confirmed the occurrence of all reactions: hydrolysis, imidization, aminomethylation, and intermolecular cross-linking reactions.

**Figure 10:**
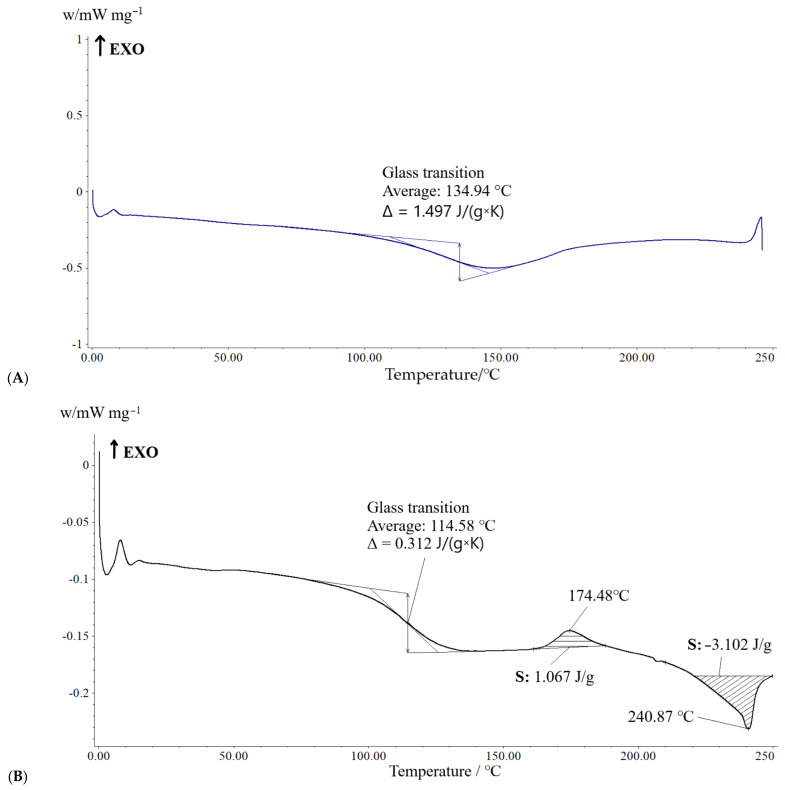
Thermogram of the LB-27 (**A**) and RB-27 (**B**) samples.

The glass transition temperatures (Tg) of the dried samples were studied by the DSC method. In Figure 10, the thermograms of LB-27 and RB-27 (after removing thermal prehistory) are shown. The temperatures of 134.94 °C, 114.58 °C, 174.48 °C, and 240.87 °C were recorded. The glass transition temperature of PAA is in the region of +190 °C, which is more than the recorded temperature. An unequal decrease in the glass transition temperature may indicate both a change in the composition of PAA in the mammary glands and an increase in the molecular weight of the polymer due to the formation of cross-links between linear PAA molecules and/or the formation of conjugated PAA molecules with high-molecular endogenous substrates. Thus, it can be assumed that within the prolonged presence of PAA gel in the mammary gland, its biochemical transformations are connected with such processes as an increase (due to cross-linking) and a decrease (due to decomposition) in the molecular weight of the polymer.

**Figure 11:**
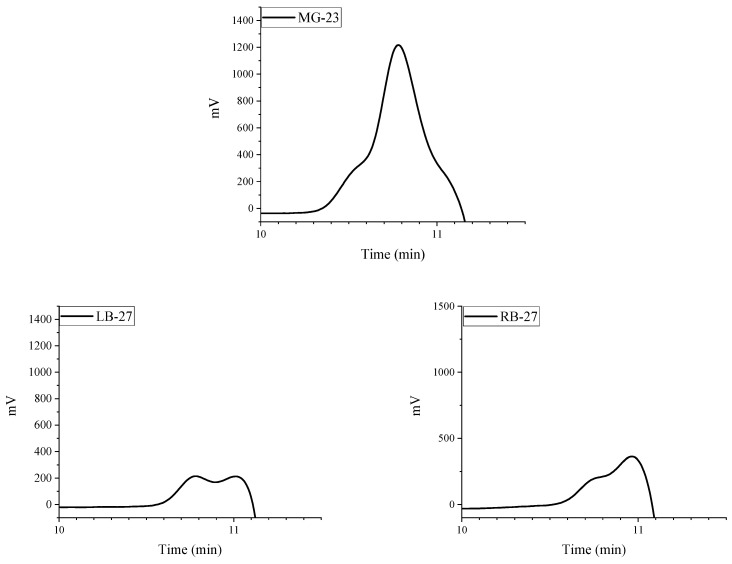
Chromatogram of separation of water-soluble extracts isolated from explants via gel-penetrating chromatography.

Further investigation of purified polymer extracts isolated from explants via gel-penetrating high-performance liquid chromatography revealed that the studied extracts consist of two polymer fractions (Figure 11), with one fraction having a lower molecular weight. This indicates a deeper process of PAA biodegradation, even with equal time of implantation, suggesting that biodegradation occurs unevenly across different parts of the mammary glands. The presence of multiple polymer fractions explains the histological variations observed at different locations in PAAG residues.

The substances isolated from acetonitrile and chloroform extracts of biopsies include:

- PAAG degradation products: N,N-dimethylacetamide (a potentially carcinogenic substance) and acetamide (an aprotic solvent);
- Eicosanoids—oxidized derivatives of polyunsaturated fatty acids are involved in a variety of processes, such as muscle tissue growth, irritation, and immune reactions to introduced toxins and pathogens, neurotransmitters and hormones: methyl ester 5,8,11,14-eicosatetraenoic acid, 2-methyleicosan, methyl ester 11,14-eicosadienoic acid;
- Products of fatty acid metabolism: 2,4-decadienal, N,N-Dimethyldodecanamide, Tetradecanamide, 9-Octadecenamide, Linoleic acid ethyl ester, 2,4-Decadienal, Hexadecanoic acid, methyl ester, 9-Octadecenoic acid (Z)-, methyl ester, Methyl tetradecanoate, Pantolactone, Ethyl Oleate, 9,12-Octadecadienoic acid, methyl ester, Dodecanamide, Pentanoic acid, 2,2,4-trimethyl-3-carboxyisopropyl, isobutyl ester, Methyl 8-methyl-nonanoate, Tetradecanoic acid, 12-methyl-, methyl ester, 10-Octadecenoic acid, methyl ester, Citric acid, triethyl ester;
- Squalene (characterized by antioxidant, cardioprotective, and anti-carcinogenic activity, as well as anti-inflammatory properties [27]);
- Phosphates, which accompany an inflammatory reaction, can serve as a source of calcinate formation (calcium phosphate).
- Cholesterol.

In silylated acetonitrile and chloroform extracts from the LB-27 and RB-27 samples, in addition to previously identified components, propionamide was also detected, which can be attributed to PAAG biodegradation products. Returning to the results of the histological assessment, we observed pronounced deformation of adipocytes. When stimulated, adipocytes release fatty acids and glycerol. The carboxyl groups of fatty acids and hydroxyl groups of glycerol readily react with amide groups present in the degradation products of PAAG, resulting in the formation of eicosanoids and metabolites of fatty acids. This process involves destructive chemical transformations such as hydrolysis, immidization, and aminomethylation, as well as intermolecular cross-linking leading to the formation of three-dimensional structures among PAAG residues.

## 3. Materials and Methods

### 3.1. Materials

The following gel masses removed after breast surgical interventions were investigated in this study:

- MG-23: Gel implanted in the mammary glands for 23 years.
- LB-27: Gel from the left breast implanted for 27 years (contains dense infiltrate inclusions).
- RB-27: Gel from the right breast implanted for 27 years.

Due to the absence of information regarding the original polyacrylamide gels, the authors are unable to compare the studied samples with control samples.

Samples for research were taken from various locations of the withdrawn gels. Isolation and purification of the water-soluble fraction from PAA explants were carried out according to the following procedure: The gel mass was carefully mixed with a spatula until a homogeneous mass was formed. After that, 12.0 g of gel suspension was taken (A200S analytical scales, Sartorius, Göttingen, Germany), to which 100 mL of distilled water was added. The resulting mixture was stirred for 4 h on a magnetic stirrer at a temperature of 60 °C. The separation of insoluble components of the solution was carried out by filtration through double-layer gauze (vacuum filtration apparatus, Sartorius, Göttingen, Germany). The filtrate was centrifuged at 4000 rpm for 10 min (laboratory centrifuge Hermle Z 300, Hermle Labortechnik, Wehingen, Germany). To remove the residue of fatty components, 20 mL of hexane (chemically pure) was added to the resulting aqueous extract. The resulting mixture was stirred for 10 min in the orbital shaker at 300 rpm. After delamination of the mixture, the upper organic layer was discarded. The collected water fraction was dried at 50 °C (ED 23 drying cabinet, BINDER, Tuttlingen, Germany; SHS-80-01 SPU drying cabinet, Smolenskoye SKTB SPU, Smolensk, Russia). 50 mL of ethanol (chemically pure) was added to the dried residue and stirred for 30 min on a magnetic stirrer at a temperature of 30 °C. After that, the solvent was removed, and the residue was dried at 50 °C to a constant weight.

### 3.2. Characterization Methods

Changes in thermophysical transitions were studied by means of Differential Scanning Calorimetry—DSC 214 Polyma (NETZSCH, Selb, Germany). A small amount (5–10 mg) of a sample was taken for analysis and heated from +10 to +250 °C at a rate of 10 °C/min in a nitrogen atmosphere in aluminum perforated crucibles. The temperature measurement error was ±1 °C.

An IR-Fourier spectrometer (IR Spirit, Shimadzu, Kyoto, Japan) was used to analyze changes that occurred in the PAA as well as to establish the chemical composition of the detected dense inclusions in the range of 7800–350 cm^−^^1^.

For gas chromatography-mass spectrometry analysis (GC-MS), a gas chromatograph with a mass spectrometric detector, Trace GC Ultra II (Thermo Fisher Scientific, Rodano, Italy) with the automated liquid sample input system TRACE AI 1310 with a capillary column (CP-Sil88, 50 m × 0.25 mm × 0.25 microns, Agilent, Santa Clara, USA) was used. The carrier gas was argon (99.9999% purity). The flow rate of the carrier gas was 1.0 mL/min. The injected volume was 1.0 µL without flow division, the injector temperature was 250 °C. The initial temperature of the thermostat was 80 °C, the final temperature of the column was 220 °C, the rate of temperature increase was 4 °C/min, and the total analysis time was 60 min. The temperature of the MSD interface was 250 °C. The mass spectra were recorded in full scan mode in the range of 40–700 m/z with a frequency of 7874 scans per second. The registration and processing of data were carried out by means of the Thermo Xcalibur software (version 2.1). The analysis of the mass spectra was carried out using the spectral library of NIST.

5.0 mL of solvent (acetonitrile or chloroform) were added to 2.0 g of biopsy sample. The mixture was incubated at 37 °C for 24 h, then the extracts were filtered using a syringe nozzle—a PTFE filter of 0.22 µm. The resulting extracts were divided into two parts. The first part—the initial extract (1.0 mL) from the appropriate solvent—was directly used for the GC-MS analysis. The second part—the organic layer from the extracts (1.5 mL) after filtration through a PTFE syringe filter—was transferred to a chromatographic vial and evaporated in the vacuum chamber to a dry residue. 50 µL of a silylating reagent were added to the dry residue in the vial—a mixture of BSTFA+TMCS (99:1, LB97455 33149-U, Supelco, Bellefonte, USA), kept in a thermostat at 80 °C for 60 min. After cooling, 200 µL of ethyl acetate were added to the vial, centrifuged for 10 min in microcentrifuge tubes, and after that, it was transferred to a chromatographic vial for analysis.

For High Performance Liquid Gel Penetrating Chromatography analysis, an LC 20AD liquid chromatograph with an RID 20A refractometric detector (Shimadzu, Kyoto, Japan) was used. The analysis was performed according to the methodology of the “Shodex” company [28]. The dry samples were pre-dissolved in water for 24 h at room temperature to reach a final concentration of 100 micrograms/mL. After that, the samples were centrifuged at 10,000 rpm for 5 min. The analysis was performed on a column Shodex^TM^ OHpak SB-806M HQ (8.0 × 300 mm) with a particle size of 13 µm. The solution of 0.1 M NaCl in distilled water was used as an eluent, separated at 30 °C at a flow rate of 1 mL/min. The sample volume was 20 µL.

The histochemical analysis of pathological breast material was performed using standard methods based on paraffin staining with hematoxylin, eosin, and Mallory.

The mineralization of the concretions included in the explants was performed at 60 °C for 6 h using a muffle furnace (EKPS-10, Smolenskoye SKTB SPU, Smolensk, Russia).

The determination of moisture content in the PAA explants was carried out by the gravimetric analysis method. The samples were dried at 105 °C for 6 h until a constant weight was reached, controlled by the analytical scales SARTORIUS A 200S (Germany). Drying chamber BINDER ED 23 (BINDER, Tuttlingen, Germany) was applied in this study. After drying, the mass of the dry residues was determined.

## 4. Conclusions

After 27 years since implantation, the presence of polyacrylamide gel residues in the injected materials has been confirmed through physico-chemical methods. Our investigation has revealed potential processes of PAA gel biotransformation, uncovering destructive chemical transformations such as hydrolysis, imidization, and aminomethylation, as well as intermolecular cross-linking leading to the formation of three-dimensional structures.

Remarkably, the biodegradation of PAA varies significantly across different mammary gland regions within a single patient. Gel-penetrating high-performance liquid chromatography has demonstrated varying proportions of polymer fractions, highlighting this uneven biodegradation process.

By means of the GC-MS analysis, we identified PAA biodegradation products, including N,N-dimethylacetamide, propionamide, acetamide, and inflammatory response mediators like eicosanoids. The coexistence of PAA gel residues and the gel’s decomposition products in the injected material suggests cyclic biotransformation processes. This cyclic nature perpetuates the presence of aggressive, toxic low-molecular-weight degradation products in the body, thereby inciting chronic inflammation.

The presence of both the PAA gel residues and its decomposition products in the injected material suggests cyclic biotransformation processes. This cyclic nature perpetuates the presence of aggressive, toxic low-molecular-weight degradation products in the body, thereby inducing chronic inflammation.

Despite the polyacrylamide content in the gels remaining stable at approximately 2%, our findings indicate that biotransformation predominantly persists. These insights shed light on the intricate dynamics of the PAA gel biotransformation, emphasizing the ongoing risk posed by its degradation products and the potential for chronic inflammatory responses.

## Figures and Tables

**Table 1:** Types and absorption ranges of characteristic vibrations identified in the IR spectra.

Types of Oscillation	The Wave Number ν, cm^−1^
Intramolecular hydrogen bonding of polyatomic alcohols, amines, and valence vibrations N-H in amino acids	3450
Wide-band, intra-complex compounds, intramolecular hydrogen bonds	3280
-CH_2_-—valence vibrations	2930–2800; 1440–1400
-COOH—valence vibrations	1760
-CO-NHR—valence vibrations	1640–1540
-C=O—valence vibrations	1640
-CO-N=—valence vibrations in tertiary amines	1650
Secondary amines in imidization reactions	1580
C=O valence symmetric and asymmetric vibrations in RCOO-. Dimers –COOH: plane deformation vibrations -OH and valence vibrations of C-O dimers of carboxyl groups	1405
-CN	1350–1210
-C=O,-C-O- valence vibrations in RC(=O)-OH	1262
-C-O-C-	1050

## Data Availability

Manuscript data are available upon an official, reasonable request to corresponding author.
